# Supplementary material for: The Role of Cytokine-Inducible SH2 Domain-Containing Protein (CISH) in the Regulation of Basal and Cytokine-Mediated Myelopoiesis
Source: Int J Mol Sci. 2023 Aug 14;24(16):12757. doi: 10.3390/ijms241612757 (PMC10454631; doi:10.3390/ijms241612757)
Supplement: Supplementary file 1 [file ijms-24-12757-s001.zip › ijms-2501733-supplementary.pdf]

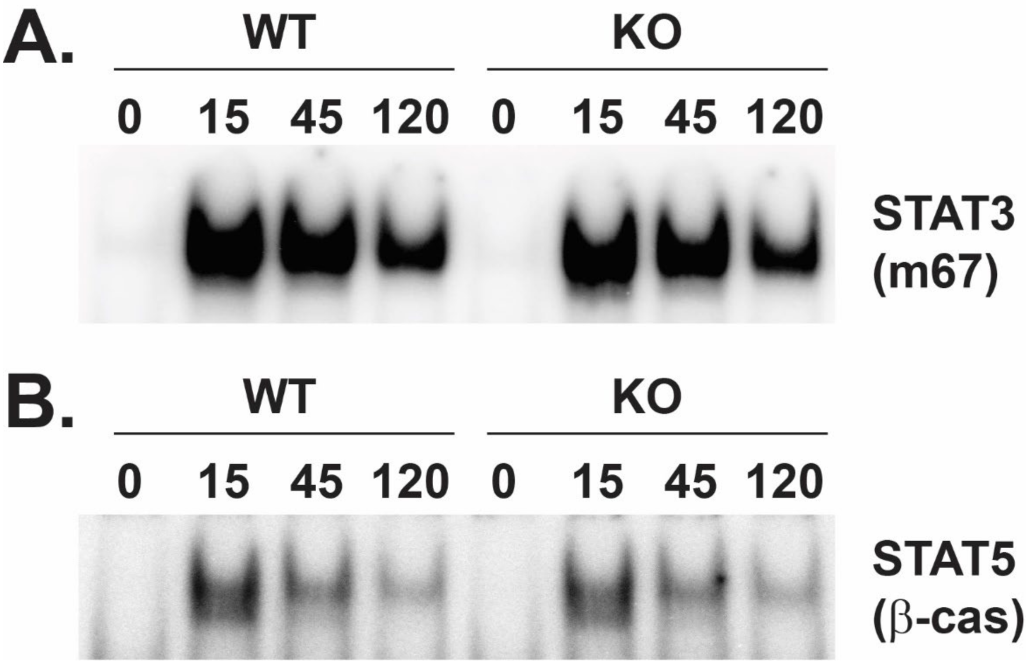

Figure S1: CISH ablation does not impact G-CSF-induced STAT activation.

Bone marrow cells (1×10^6) from *Cish*$^{+/+}$ (WT) and *Cish*$^{-/-}$ (KO) mice were stimulated ex vivo with G-CSF for the indicated time (min) and nuclear extracts prepared for analysis by EMSA using probes for STAT3 (m67) (A) and STAT5 (β-cas) (B) as indicated.

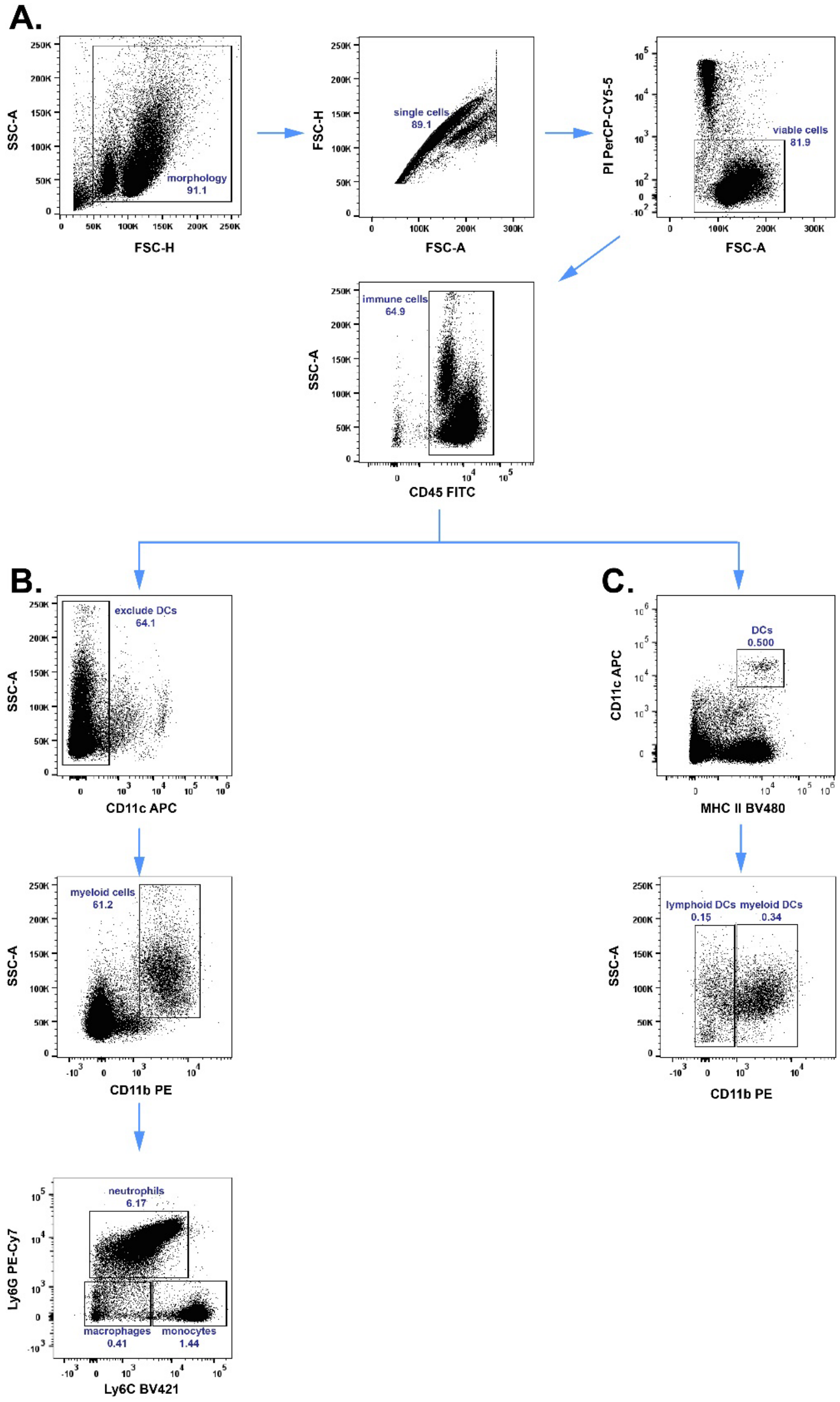

Figure S2: Flow cytometry gating strategy used for myeloid cell phenotyping.

Strategy used to quantify the indicated myeloid cell populations in bone marrow and spleen, showing representative plots from a *Cish*$^{+/+}$ Balb/c mouse. Total cells were first gated on the basis of morphology on a side scatter area (SSC-A) versus forward scatter height (FSC-H) plot, with single cells then gated on a FSC-H versus forward scatter area (FSC-A) plot, viable cells on a propidium iodide (PI-PerCP-CY5-5) versus FSC-A plot and immune cells identified as CD45.2^{+} on a SSC-A versus CD45.2 (CD45-FITC) plot (A). Neutrophils, macrophages and monocytes were identified by excluding CD11c^{-} cells on a SSC-A versus CD11c (CD11c-APC) plot, and selecting CD11b^{+} cells on a SSC-A versus CD11b (CD11b-PE) plot, with individual lineages identified by gating on a Ly6C (Ly6C-PE-Cy7) versus Ly6G (Ly6G-BV421) plot as indicated (B). Dendritic cells (DCs) were identified by gating double-positive cells on a CD11c versus MHC class II (MHCII-BV480) plot, with lymphoid and myeloid DCs identified by gating on a SSC-A versus CD11b plot as indicated (C).
